# Hereditary Pancreatic Cancer: A Retrospective Single-Center Study of 5143 Italian Families with History of BRCA-Related Malignancies

**DOI:** 10.3390/cancers11020193

**Published:** 2019-02-07

**Authors:** Angela Toss, Marta Venturelli, Eleonora Molinaro, Stefania Pipitone, Elena Barbieri, Isabella Marchi, Elena Tenedini, Lucia Artuso, Sara Castellano, Marco Marino, Enrico Tagliafico, Elisabetta Razzaboni, Elisabetta De Matteis, Stefano Cascinu, Laura Cortesi

**Affiliations:** 1 Department of Oncology and Hematology, University Hospital of Modena, 41124 Modena, Italy; martaventurelli@msn.com (M.V.); ele.molinaro.89@gmail.com (E.M.); stefania.pipitone88@gmail.com (S.P.); barbieri.elena@aou.mo.it (E.B.); marchi.isabella@policlinico.mo.it (I.M.); elisabetta.razzaboni@gmail.com (E.R.); stefano.cascinu@unimore.it (S.C.); hbc@unimore.it (L.C.); 2 Centre for Genome Research, University of Modena and Reggio Emilia, 41124 Modena, Italy; elena.tenedini@unimore.it (E.T.); lucia.artuso@unimore.it (L.A.); enrico.tagliafico@unimore.it (E.T.); 3 Clinical Genomics Laboratory, Department of Laboratory Medicine and Pathology, University Hospital of Modena, 41124, Modena, Italy; marcomarino83@gmail.com; 4 Clinical and Experimental Medicine PhD Program, University of Modena and Reggio Emilia, 41124 Modena, Italy; sara.castellano@unimore.it; 5 Department of Oncology, Vito Fazzi Hospital, 73100 Lecce, Italy; dr.dematteis.elisabetta@gmail.com

**Keywords:** BRCA genes, pancreatic cancer, genetic testing, hereditary cancer, homologous recombination

## Abstract

The identification of *BRCA* mutations plays a crucial role in the management of hereditary cancer prevention and treatment. Nonetheless, *BRCA*-testing in pancreatic cancer (PC) patients is not universally introduced in clinical practice. A retrospective analysis was conducted, firstly, to evaluate the rate of *BRCA*-positive families among those presenting a family history of PC besides breast and/or ovarian cancer. Secondly, the relationship between *BRCA* pathogenic variants and PC risk was evaluated. Finally, the characteristics of PC developed in *BRCA* families were described. Among 5143 family trees reporting breast and/or ovarian cancer cases, 392 showed a family history of PC. A total of 35 families (24.5% selected by the Modena Criteria and 21.3% by the NCCN Criteria) were positive to *BRCA* testing. Among the *BRCA1* mutations, 36.8% were found within a region defined by c.3239–c.3917, whilst 43.7% of *BRCA2* mutations were located within c.7180–c.8248. This study confirmed that an increase in the rate of positive tests in families with PC when associated to breast and/or ovarian tumors. Moreover, this analysis indicated two possible Pancreatic Cancer Cluster Regions that should be verified in future research. Finally, PC in families with breast and/or ovarian cancer history, particularly in *BRCA* families, were diagnosed at younger age and showed better one-year overall survival.

## 1. Introduction

The *BRCA1* and *BRCA2* genes encode for proteins involved in tumor suppression. Particularly, the *BRCA* genes are involved in the repair of DNA double-stranded breaks (DSBs) through the mechanism of homologous recombination (HR) [1]. Currently, hundreds of different mutations have been identified in both *BRCA1* and *BRCA2* genes, and individuals carrying these mutations in parental germline cells showed an increased susceptibility to several solid cancers. Tumors developing in these individuals are classified as hereditary cancers. Based on this information, when an individual is suspected of being at risk of hereditary cancer according to his personal and family cancer history, a genetic counseling and possibly a genetic testing should be offered [2,3,4].

Overall, *BRCA1/2* mutation carriers present an increased risk for breast cancer (52–72% in *BRCA1*, 45–84% in *BRCA2*), ovarian cancer (39–63% in *BRCA1*, 11–27% in *BRCA2*), prostate cancer (3.4-fold increased risk in *BRCA1*, 8.6-fold increased risk in *BRCA2*), and pancreatic cancer (1–3% in *BRCA1*, 2–7% in *BRCA2*) [5,6,7,8,9]. Moreover, an increased frequency of other malignancies, such as melanoma and other gastro-intestinal tumors, has been reported in families with mutations in the *BRCA2* gene [7]. As a result, several National and International Guidelines published *BRCA* testing criteria for the selection of the best candidates for genetic testing. The American National Comprehensive Cancer Network (NCCN) Guidelines present one of the highest sensitivity in BRCA carrier detection [10,11], and include personal and family history of breast, ovarian, prostate, and pancreatic cancers among the BRCA testing criteria, as reported in Table 1. On the other hand, according to the Italian Association of Medical Oncology (AIOM) Guidelines, which reflect the Modena Criteria [12,13,14] listed in Table 2, the Italian National Health Service provides free *BRCA* diagnostic tests exclusively to breast and ovarian cancer patients and healthy individuals with an estimated risk of carrying a *BRCA* mutation ≥ 40%. All the Italian criteria are included in the NCCN Guidelines.

The identification of a mutation in *BRCA* genes plays a crucial role in the management of hereditary cancer prevention, diagnosis, and treatment. In healthy *BRCA* carriers, the detection of a mutation may justify more intensive and personalized surveillance programs [15], chemo-preventive approaches [16], and prophylactic surgery [17] that would not otherwise be justified by family history alone. On the other hand, the identification of a mutation in patients already affected by cancer may provide the fundamental knowledge of the pathogenesis of these tumors, thereby guiding treatment choices. In particular the impairment of the HR pathway is thought to confer major sensitivity to platinum agents, and the inhibition of poly (ADP-ribose) polymerase (PARP) by the use of PARP inhibitors, resulting in the persistence of DNA damage and in cell cycle arrest [1,18]. Despite these considerations, BRCA-testing in pancreatic cancer patients and their families is not universally introduced in clinical practice. Due to the high cost of genetic tests, especially in those countries where genetic testing is freely available, BRCA1/2-testing has traditionally been restricted to individuals with a high risk of being a carrier.

The purposes of our retrospective analysis were: Firstly, to evaluate the rate of BRCA-positive families among those presenting a family history of pancreatic cancer besides breast and/or ovarian cancer; secondly, to evaluate whether a relationship exists between BRCA pathogenic variants and pancreatic cancer risk in the family and, finally, to explore the characteristics of pancreatic cancers developed in BRCA positive families.

## 2. Results

### 2.1. Family Characteristics

**Figure 1:**
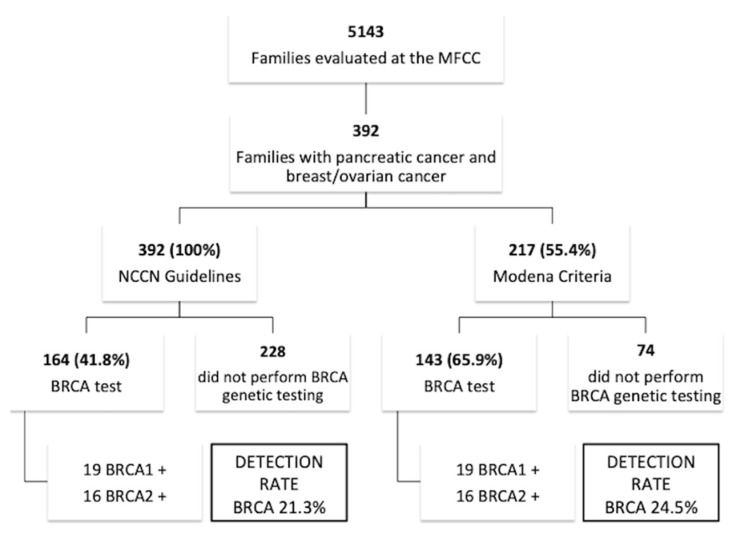
Study flow chart.

From January 1997 to May 2017, 5143 families with a positive family history for breast and/or ovarian cancer were counseled and taken in charge by the Modena Family Cancer Clinic (MFCC). Among these, 5143 family trees stored in our archives, 392 (7.6%) also reported a family history of pancreatic cancer (Figure 1). In particular, 6 families (one of which *BRCA1* positive) showed three pancreatic cancer cases, and 31 families (4 of which *BRCA1* positive and 2 of which *BRCA2* positive) showed two pancreatic cancer cases.

All these families (100%) were candidate to *BRCA* testing according to the NCCN Guidelines 2019, whereas only 217 (55.4%) of these families were candidate to *BRCA* testing according to the Modena Criteria. Among families selected using the NCCN Criteria (Table 1), 69 (17.6%) families showed an early onset breast cancer (diagnosis at ≤ 35 years) in the pedigree, 13 (3.3%) had a male breast cancer, 71 (18.1%) showed at least one case of ovarian cancer, while in 86 (21.9%) families pancreatic cancer was associated to only one sporadic breast cancer (diagnosis at > 40 years). On the other hand, among families selected by the Modena Criteria, 69 (31.8%) families showed an early onset breast cancer (diagnosed at ≤ 35 years) in the pedigree, 13 (5.9%) had a male breast cancer, 70 (32.2%) showed at least one case of ovarian cancer, while pancreatic cancer was never associated to sporadic breast cancer (diagnosed at > 40 years).

One hundred and 43 (65.9%) families selected according to the Modena Criteria were subject to BRCA testing, and 19 *BRCA1* mutations and 16 *BRCA2* mutations were identified (detection rate 24.5%). On the other hand, 164 (41.8%) families that were selected with the NCCN Criteria were subject to BRCA testing and showed a detection rate of 21.3%.

### 2.2. BRCA Pathogenic Variants and Pancreatic Cancer Risk

**Figure 2:**
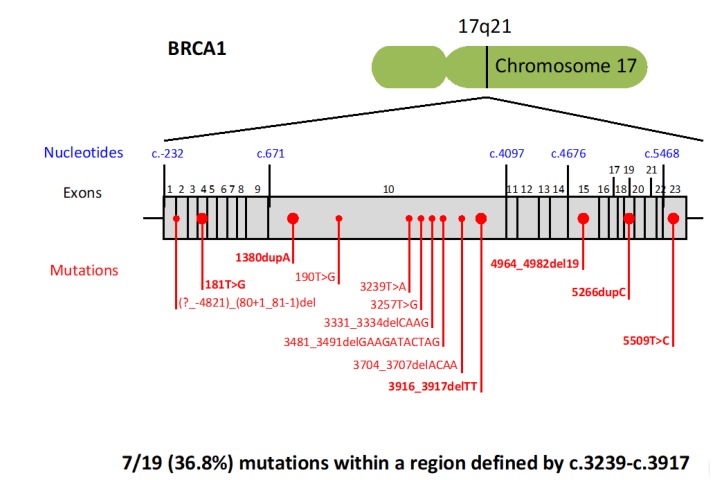
Distribution of *BRCA1* pathogenic variants. The mutations detected twice are shown in bold.

**Figure 3:**
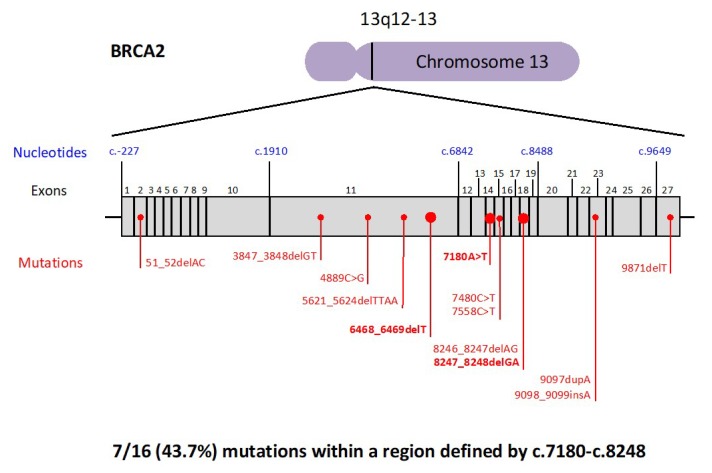
Distribution of *BRCA2* pathogenic variants. The mutations detected twice are shown in bold.

The *BRCA* mutations detected in families with history of pancreatic cancer are reported in Figure 2 and Figure 3. Ten out of 19 (52.6%) *BRCA1* mutations were located in EXON10. On the other hand, the most mutated exon in BRCA2 was EXON11 (5 out of 16, 31.2%), followed by EXON18 (3 out of 16, 18.7%). In particular, 7 out of 19 (36.8%) *BRCA1* mutations were found within a region defined by c.3239–c.3917 (Figure 2), while 7 out of 16 (43.7%) *BRCA2* mutations were located within the interval c.7180–c.8248 (Figure 3). Finally, the most common *BRCA1* mutation types were frame-shift mutations (9 out of 19, 47.3%), followed by missense mutations (5 out of 19, 26.3%). With regards to BRCA2, the most frequent mutation types were frame-shift mutations (10 out of 16, 62.5%), followed by nonsense mutations (5 out of 16, 31.2%).

### 2.3. Characteristics of Pancreatic Cancers Developed in BRCA Families

The average age for pancreatic cancer diagnosis was 66 (range 20–94) in the overall study population, 65 (46–85) in the 19 *BRCA1* positive families and 66 (range 49–80) in the 16 *BRCA2* positive families. Therefore, pancreatic cancer patients in our study population were diagnosed at younger ages than the general population, where the average age for diagnosis is 70, according to the Surveillance, Epidemiology and End Results (SEER) program [19] (Table 3).

The one-year OS rate was 42% in the overall study population, 42.8% in *BRCA1* positive families and 61.5% in *BRCA2* positive families. Therefore, pancreatic cancer patients in our study population showed a higher one-year OS rate than the general population, where the rate is 23% according to the Italian Cancer Registry [20]. Finally, 5-year OS rate was 6.6% in the overall study population, 7.1% in *BRCA1* positive families and 0% in *BRCA2* positive families. Therefore, pancreatic cancer patients in our study population showed lower 5-year OS rate than the general population where the rate is 8.1% according to the Italian Cancer Registry [21] (Table 3).

## 3. Discussion

Pancreatic ductal adenocarcinoma has a poor prognosis, with a 5-year survival rate of only 5% [22]. Approximately 4% of all patients with pancreatic ductal adenocarcinoma have an underlying gene defect, with *ATM*, *BRCA1/2*, and *PALB2* being the most commonly affected genes [23,24,25]. Overall, only 3–10% of patients have a positive family history for this cancer [26]. In particular, recent studies found deleterious germline mutations in pancreatic cancer predisposition genes in 3.9–7.7% sporadic pancreatic adenocarcinomas, and around 45% of these mutations were in one of the *BRCA* genes [27,28,29]. Interestingly, in these studies germline mutations were identified in patients with pancreatic cancer without a significant family history of cancer. This highlights the limitations of many current genetic testing criteria for patient selection. Following a systematic review of the literature published from January 1998 to June 2018, a recently published American Society of Clinical Oncology (ASCO) Provisional Clinical Opinion states that all patients diagnosed with pancreatic adenocarcinoma should be assessed for the risk of hereditary syndromes, known to be associated with an increased risk for pancreatic adenocarcinoma [30].

Currently, surgical resection is the only potentially curative treatment for pancreatic ductal adenocarcinoma, but in approximately 80% of symptomatic patients at diagnosis, the tumor is already unresectable. Improvements in the resectability of tumors requires the detection of tumors at an earlier stage; therefore, there is an urgent need to find effective screening programs especially for individuals at an increased family risk. Surveillance programs based on annual magnetic resonance imaging, magnetic resonance cholangiopancreatography, and/or endoscopic ultrasound, recently demonstrated an increase in the detection of pancreatic tumors at a resectable stage in *CDNK2A* mutation carriers [31]. Therefore, the identification of a mutation in pancreatic cancer susceptibility genes may justify more intensive and personalized surveillance programs [32].

In an attempt to determine the incidence and the characteristics of *BRCA*-associated pancreatic cancers, we reviewed our institution’s experience. A high rate of germline *BRCA1* and *BRCA2* mutations was detected in our retrospective analysis of 392 families with pancreatic cancer associated with breast and/or ovarian cancer. In particular, the detection rate was 21.3% when the NCCN Guidelines were applied, and 24.5% with the Modena Criteria. Overall, the detection rate of *BRCA* mutation when applying the Modena Criteria in our daily clinical experience is 17%. Therefore, our retrospective study confirmed an increased rate of positive *BRCA1/2* test in families with pancreatic cancers when associated to breast and/or ovarian tumors. Moreover, although a lower rate of families were tested in the NCCN group, the Modena Criteria seems to be more cost-effective than the NCCN Guidelines in the identification of individuals carrying the mutation. Interestingly, our analysis also shows that only 7 out of 35 (20%) *BRCA*-associated pancreatic cancer patients presented a family history of pancreatic cancer. While the approval of PARP inhibitors in pancreatic cancer treatment, and the extension of genetic testing to all candidate pancreatic cancer patients for those treatments is still underway, our findings support the introduction of pancreatic cancer among the testing criteria, at least when associated to family history of breast or ovarian cancer.

The *BRCA* mutations, detected in our families with a history of pancreatic cancer, were more frequently located in the longest exon of each gene, EXON10 of the *BRCA1* gene and in the EXON11 of the *BRCA2* gene. Interestingly, we identified two nucleotide intervals presenting a higher incidence of mutations: The *BRCA1* region is defined by c.3239–c.3917 and the *BRCA2* region is located within c.7180–c.8248. These intervals correspond to, neither the previously identified Breast Cancer Cluster Regions (BCCR), nor the Ovarian Cancer Cluster Regions (OCCR) of *BRCA1*, and only marginally overlap with the BCCRs and the OCCRs of BRCA2 [33]. As for breast and ovarian cancer, it is likely that mutations in a specific gene region may influence the risk and the characteristics of pancreatic cancer that is developed by *BRCA* mutation carriers. Our findings indicated two possible Pancreatic Cancer Cluster Regions that should be verified in larger cohort of *BRCA*-associated pancreatic cancer patients.

As for the *BRCA*-associated breast and ovarian cancer, *BRCA* carriers that were affected by pancreatic cancer were demonstrated to be optimal candidates to platinum-based therapy and PARP inhibitors [34,35,36,37,38]. For this reason, along with the early diagnosis provided by the surveillance programs, the identification of a *BRCA* mutation in pancreatic cancer patients should assume a central role in the improvement of the outcome for these patients.

According to our results, pancreatic cancers in families with breast and/or ovarian cancer history are diagnosed at a younger age and show better one-year OS than those developed in the general population. These results may be justified by the presence of germ-line mutations that predispose these families to hereditary pancreatic, breast, and ovarian tumors. Interestingly, in line with previous literature [37,39], these findings are also confirmed in the *BRCA* families. Particularly, the age for pancreatic cancer diagnosis in *BRCA1* families is significantly lower than in *BRCA2* families and general populations, whereas short-term prognosis for patients in *BRCA2* families is significantly better than for other groups of patients. Similar results were previously observed in the recently published POSH study [40], in which BRCA-associated triple-negative breast cancer showed a better survival rate over the first few years after diagnosis. This early survival advantage was also previously observed among *BRCA* patients with ovarian cancer [41]. This short-term survival advantage might reflect greater sensitivity of *BRCA*-mutant cancers to chemotherapy, particularly in pancreatic and ovarian cancers, that are usually treated with platinum-based regimens in the first line setting. However, his survival advantage is likely to be lost lost because of the treatment resistance mechanisms that can develop, such as the occurrence of secondary *BRCA* mutations that restore the BRCA function and HR activity.

There are certainly some limitations to our retrospective analysis. It should be noted that in our study, the cancer family history was collected from the anamnesis of women who accessed the MFCC, because of their breast and/or ovarian family history. Therefore, a bias in data collection could be present, since there is a chance, that some cancer cases and their outcome, were wrongly reported by patients’ relatives and, therefore, misclassified. For the same reason, we do not have any information regarding surgical and medical treatments undergone by these pancreatic cancer patients. Finally, since most pancreatic cancer patients were not alive at the time of genetic counseling, it was not possible to verify which pancreatic cancer patients in *BRCA* positive families were actually *BRCA* carriers.

## 4. Material and Methods

### 4.1. Study Population and Design

Since 1995, the MFCC takes charge of women with a family history of breast and/or ovarian cancer. According to the Modena Criteria [12,13] and, more recently, by the Tyrer-Cuzick model [17], women are classified in risk categories and are included in personalized surveillance programs. Moreover, women or their relatives who meet the Modena Criteria for genetic testing can undergo the BRCA test and, according to the result, may access risk-reducing surgeries [18], chemo-preventive studies [16] or more intensive surveillance programs [15]. During pre-test counseling, family and personal history of cancer is collected and a family tree is drawn. Finally, after the post-test counseling, a copy of all patients’ documents and reports are stored in the archive of the MFCC.

For the purposes of our study, we retrospectively identified families with at least one pancreatic cancer reported in the family tree and registered them in our archive. Then, each family was analyzed according to the NCCN *BRCA* testing Criteria and the Modena Criteria, in order to evaluate the number of candidate families to genetic testing in each group. Finally, we evaluated the rate of positive tests in each group, defining the detection rate of *BRCA* mutations according to the different criteria applied.

In addition, we looked at the *BRCA* pathogenic variants detected, to investigate a possible association between the gene region affected by mutation and pancreatic cancer risk. Finally, we analyzed age at diagnosis of pancreatic cancer, the one-year and 5-year overall survival rate in families with BRCA mutation compared to the general population.

### 4.2. BRCA Testing Procedures

Before 2014, the genetic testing of *BRCA1* and *BRCA2* genes in our institution was carried out by direct Sanger sequencing, whilst after 2014, it was performed using next generation sequencing (NGS). With both methods, the molecular test was performed on genomic DNA, isolated from fresh peripheral blood samples, encompassing the entire coding region and adjacent intronic splice-site consensus sequences of *BRCA* genes. The NGS workflow benefited from the use of the Ion AmpliSeq^TM^ (Thermo Fisher Scientific, Waltham, MA, USA) technology that was handled initially with a semi-automated and subsequently, with a fully automated procedure for multiplex PCR-based library preparation and sequencing on the Ion Torrent platforms (Thermo Fisher Scientific, Waltham, MA, USA). Sanger sequencing was routinely performed to validate candidate mutations, as long as multiplex ligation probe amplification (MLPA, MRC-Holland, Amsterdam, the Netherlands) was carried out to detect copy number variations. Sequences alignment, base calling, variants filtering, and annotation process took advantage of the Torrent Software Suite (Thermo Scientific) and of a custom designed bio-informatic pipeline, as described in previously published works [42,43].

## 5. Conclusions

Our study suggests the possibility of an improvement in the National and International BRCA testing criteria by including pancreatic cancer cases among the criteria. Further research should be directed to evaluating whether the detection of a *BRCA* mutation could improve the outcome of pancreatic cancer patients, by the introduction of prevention strategies and tailored treatments. Moreover, since the risk of pancreatic cancer increases 10-fold in *BRCA* carriers, more effort is needed to identify which mutations are associated with a significantly increased risk for this tumor.

## Figures and Tables

**Table 1:** The NCCN BRCA testing criteria (NCCN Guidelines Version 2.2019).

**Breast Cancer Diagnosed ≤ 45 Years**
OC, fallopian tube or primary peritoneal cancer at any age.
Male breast cancer.
Triple negative BC diagnosed ≤ 60 years.
BC diagnosed 46–50 years with a second BC primary at any age.
BC diagnosed 46–50 years with ≥ 1 close relative with BC or prostate cancer (GS ≥ 7) or with unknown or limited family history.
BC diagnosed at any age with ≥ 1 close relative with BC ≤ 50 years or OC or male BC or metastatic prostate cancer or pancreatic cancer.
BC diagnosed at any age with ≥ 2 additional diagnosis of BC at any age in patient and/or in close blood relatives.
Personal history of BC or prostate cancer (GS ≥ 7) with Ashkenazi Jewish ancestry.
Pancreatic cancer.
Metastatic prostate cancer.
Prostate cancer (GS ≥ 7) at any age with ≥1 close blood relative with OC at any age or pancreatic cancer or metastatic prostate cancer or BC ≤ 50 years.
Prostate cancer (GS ≥ 7) at any age with ≥ 2 close blood relatives with BC or prostate cancer (any grade).
BRCA 1/2 pathogenic/likely pathogenic mutation detected by tumor profiling of any tumor type in the absence of germline pathogenic/likely pathogenic variant analysis.
Regardless of family history, some individuals with a BRCA-related cancer may benefit from genetic testing to determinate eligibility for targeted treatment.
An individual who does not meet the other criteria but with ≥ 1 first- or second-degree blood relative meeting any of the above criteria. The significant limitations of interpreting test results for an unaffected individual should be discussed.

BC: Breast Cancer; OC: ovarian cancer; GS: Gleason Score; Close blood relatives include first-, second- and third- degree relatives on same side of family.

**Table 2:** The Modena Criteria (AIOM Guidelines 2018).

**BC and OC Diagnosed in The Same Patient**
OC, fallopian tube or primary peritoneal cancer (excluded mucinous and borderline) at any age.
Male Breast Cancer.
Triple negative BC diagnosed ≤60 years.
BC diagnosed ≤ 35 years.
At least 1 BC and at least 1 OC.
At least 2 first-degree blood relative with BC, at least one diagnosed ≤ 40 years or bilateral.
Healthy individuals with an estimated risk of carrying a BRCA mutation ≥ 40%, calculated with the BRCAPro risk calculator (Version CaGene6)

BC: Breast Cancer; OC: ovarian cancer.

**Table 3:** Pancreatic cancer characteristics in our sample and in the general population [20,21,22].

	Overall Study Population (392 patients)	BRCA1 Families (19 patients)	BRCA2 families (16 patients)	General Population (Cancer Registries)
Age at Pancreatic Cancer Diagnosis	65.8 (20–94) (31 unknown)	65.2 (46–85) (3 unknown)	66.3 (49–80) (1 unknown)	70 [18]
one-year OS	42% (59 unknown)	42.8% (5 unknown)	61.5% (3 unknown)	23% [19]
5-year OS	6.6% (59 unknown)	7.1% (5 unknown)	0% (3 unknown)	8.1% [20]
